# Electrical and Structural Atrial Remodeling Identifies a High-Risk Phenotype in Embolic Stroke of Undetermined Source

**DOI:** 10.1016/j.jacadv.2026.102990

**Published:** 2026-07-09

**Authors:** Niklas Bach, Louisa Bauermeister, Lisann Böhnke, Alexander Zuk, Stephan Salmen, Adnan Labedi, Vasileios Gkizas, Alanna Ebigbo, Ralf Gold, Arash Haghikia, Michael Gotzmann

**Affiliations:** a Department of Cardiology and Rhythmology, University Hospital St Josef Hospital Bochum, Germany; b Department of Neurology, University Hospital St Josef Hospital Bochum, Germany; c Department of Internal Medicine, University Hospital St Josef Hospital Bochum, Germany

**Keywords:** atrial cardiomyopathy, embolic stroke of undetermined source, advanced interatrial block, electrocardiography, NT-proBNP

## Abstract

**Background:**

Embolic stroke of undetermined source (ESUS) carries a substantial recurrence risk, yet anticoagulation has not proven superior to antiplatelet therapy in unselected patients. Atrial cardiomyopathy, reflecting electrical and structural atrial remodeling, may identify a high-risk ESUS subgroup.

**Objectives:**

The purpose of this study was to characterize the extent of electrical and structural atrial remodeling in patients with ESUS and to evaluate its association with adverse cardiovascular outcomes during follow-up.

**Methods:**

In this prospective single-center study, 103 consecutive ESUS patients and 123 age- and sex-matched controls without known cardiac disease underwent clinical evaluation, NT-proBNP measurement, 12-lead electrocardiogram, and transthoracic echocardiography. Patients were followed for ≥12 months. The primary endpoint was a composite of all-cause death, recurrent stroke, transient ischemic attack, myocardial infarction, or newly diagnosed atrial fibrillation. Independent predictors were determined using multivariable Cox regression.

**Results:**

ESUS patients exhibited higher NT-proBNP, more frequent advanced interatrial block, and impaired left atrial function compared with controls. Over a median follow-up of 382 days (IQR: 366-697 days), 29 ESUS patients (29/103, 28%) reached the primary endpoint. Independent predictors were NT-proBNP >420 pg/mL, advanced interatrial block, E′ ≤9 cm/s, left atrial volume index ≥29 mL/m^2^, and left atrial ejection fraction <50%. A risk score integrating ≥3 markers identified a subgroup with markedly increased risk, in whom Kaplan-Meier estimated event-free survival at 1 year was approximately 50% (95% CI: 31.6%-68.4%).

**Conclusions:**

Multimodal assessment of electrical and structural atrial remodeling identifies a subgroup of ESUS patients with an increased risk of adverse events. These findings underscore the potential clinical relevance of atrial cardiomyopathy and may support further evaluation in future rhythm-guided or anticoagulation studies.

Ischemic stroke remains a major cause of mortality and long-term disability worldwide.^1^ A substantial proportion of events—approximately 15% to 25%—are classified as embolic stroke of undetermined source (ESUS),^2^ reflecting a presumed embolic mechanism without an identifiable cardioembolic substrate. Despite advances in diagnostic work-up and secondary prevention, ESUS patients continue to experience a considerable risk of recurrent events,^3^ highlighting the need for improved mechanistic phenotyping.

The concept of atrial cardiomyopathy (AtCM) has emerged as a potential link between atrial disease and thromboembolism beyond overt atrial fibrillation.^4^, ^5^, ^6^ Contemporary consensus documents describe AtCM as a progressive spectrum of electrical, structural, and functional atrial remodeling that may promote thromboembolism even in the absence of documented atrial fibrillation.^7^^,^^8^ Experimental and clinical data suggest that atrial substrate abnormalities—including atrial enlargement, impaired mechanical function, conduction delay, and biomarker elevation—may represent a prothrombotic atrial phenotype.

Randomized trials evaluating empirical anticoagulation in unselected ESUS populations have not demonstrated superiority over antiplatelet therapy.^9^, ^10^, ^11^, ^12^, ^13^ The ARCADIA (Atrial Cardiopathy and Antithrombotic Drugs in Prevention After Cryptogenic Stroke) trial subsequently tested anticoagulation in ESUS patients selected for predefined AtCM markers, yet did not show a reduction in recurrent stroke.^12^ These findings raise critical questions regarding the optimal definition and phenotyping of clinically relevant AtCM. It remains unclear whether currently applied diagnostic thresholds adequately capture the subset of ESUS patients with advanced atrial remodeling and persistently elevated cardiovascular risk.

Multimodal assessment integrating biomarkers, surface electrocardiogram (ECG) parameters, and echocardiographic measures may provide a more refined characterization of atrial substrate.^6^ In particular, electrocardiographic indices of interatrial conduction delay and echocardiographic markers of left atrial (LA) structural and functional remodeling are scalable tools with potential prognostic relevance.^14^

Accordingly, the present study pursued 2 objectives: first, to compare multimodal markers of AtCM between ESUS patients and age- and sex-matched controls without overt cardiac disease; and second, to determine whether a combination of electrical and structural atrial remodeling parameters identifies a high-risk ESUS phenotype associated with adverse cardiovascular outcomes.

## Methods

### Study design and population

This prospective, single-center study represents an analysis of the ARCANA study (AtRial CArdiomyopathy–prevaleNce And clinical impact), conducted through interdisciplinary collaboration between cardiology/electrophysiology, neurology, and internal medicine at St. Josef Hospital Bochum, Ruhr University Bochum.

From July 2022 to December 2024, consecutive patients with ESUS were enrolled. An age- and sex-matched control group without prior stroke or transient ischemic attack (TIA) and without manifest cardiac disease served as comparators. All participants were followed for at least 12 months. Written informed consent was obtained from all patients. The study was approved by the local ethics committee (registration number 22-7535).

### Inclusion and exclusion criteria

ESUS group: Inclusion criteria were: 1) ESUS according to current criteria^15^: imaging-confirmed nonlacunar ischemic infarction, no ≥50% stenosis in an artery supplying the infarct territory, no major cardioembolic source, and no alternative specific stroke etiology; 2) exclusion of a patent foramen ovale; 3) sinus rhythm at study inclusion; 4) age ≥18 and <90 years; 5) life expectancy >1 year; and 6) written informed consent.

Exclusion criteria were: 1) atrial fibrillation before or during hospitalization; 2) structural heart disease (eg, severe valvular disease or left ventricular ejection fraction [LVEF] <30%); 3) severe renal failure (glomerular filtration rate <15 mL/min); 4) severe hepatic disease; 5) manifest hyperthyroidism; 6) prior valve replacement or coronary artery bypass surgery; and 7) continuous pacemaker stimulation.

Control group: controls were age- and sex-matched individuals with: 1) no known or manifest heart disease (except left ventricular hypertrophy due to hypertension); and 2) no history of stroke or TIA.

General exclusion criteria (all patients): 1) severe valvular disease; 2) use of antiarrhythmic drugs; 3) prior pulmonary vein isolation or cardiac surgery; 4) continuous pacemaker stimulation or nonsinus rhythm; 5) electrical or pharmacological cardioversion within 3 months; 6) end-stage renal disease; and 7) LVEF <30%.

### Clinical and laboratory assessment

Medical history was obtained in all patients, and blood samples were collected and analyzed. Blood pressure was measured immediately before echocardiography. Twelve-lead ECGs were recorded immediately before or after echocardiography.

### ECG and echocardiography

The methodology has been described previously in the ARCANA study.^14^ Briefly, ECGs were recorded at 50 mm/s and 10 mm/mV. Two blinded investigators independently analyzed all ECGs; discrepancies were resolved by a third reviewer. P-wave parameters were assessed according to International Society of Electrocardiology recommendations.^16^ Heart rate, P-wave duration, PR interval, and QRS duration were recorded. Advanced interatrial block (IAB) was defined as P-wave duration ≥120 ms with biphasic morphology in leads II, III, and aVF (Figure 1).

**Figure 1:**
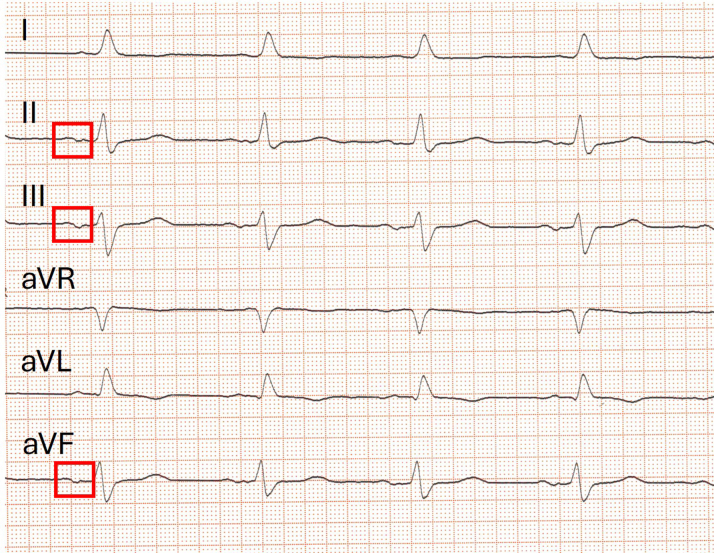
Advanced Interatrial Block on 12-Lead Electrocardiogram Representative 12-lead electrocardiogram demonstrating advanced interatrial block. The P wave is highlighted in red boxes, illustrating prolonged P-wave duration with biphasic morphology in the inferior leads, consistent with advanced interatrial block.

Transthoracic echocardiography was performed by an experienced investigator according to European Society of Cardiology guidelines^17^^,^^18^ using Vivid E9 or E95 systems (GE Healthcare, Horten, Norway). Images were archived and analyzed with EchoPAC software (GE Healthcare). The P-wave to A′ tissue Doppler interval was measured from the onset of the P wave to the peak late diastolic velocity at the lateral mitral annulus.^19^ Tissue Doppler parameters (E′, A′, S′) were obtained in the apical 4-chamber view at lateral and septal sites and averaged. Left atrial strain was assessed in apical 2- and 4-chamber views.^20^

Interobserver and intraobserver variability for ECG and echocardiographic measurements was assessed in a randomly selected subset of 25 patients from the full study cohort, demonstrating good reproducibility. Measurements were performed by 2 independent observers blinded to clinical data.

### Management of ESUS patients

All ESUS patients were treated in a comprehensive stroke unit. Diagnosis was made by experienced neurologists based on current recommendations. Treatment and secondary prevention were independent of the study protocol and included aspirin 100 mg daily. No patient received oral anticoagulation.

### Atrial fibrillation ascertainment

Atrial fibrillation was assessed as part of routine clinical evaluation in patients with ESUS. During the index hospitalization, patients underwent continuous in-hospital rhythm monitoring and standard electrocardiographic assessment. Patients with documented atrial fibrillation during hospitalization were excluded from the study cohort.

During follow-up, atrial fibrillation detection was based on clinically indicated evaluations by treating physicians, including general practitioners and cardiologists. Atrial fibrillation diagnosis was ascertained through structured follow-up information and verified by review of medical records, including physician correspondence and outpatient documentation.

No systematic prolonged ambulatory rhythm monitoring or implantable loop recorder implantation was performed as part of the study protocol.

## Outcomes

Events were defined as: 1) all-cause death; 2) recurrent stroke; 3) new TIA; 4) myocardial infarction; and 5) newly detected atrial fibrillation. The primary endpoint was the composite of these 5 events. The secondary endpoint was the composite of all-cause death and recurrent stroke.

### Follow-up

Follow-up started at the date of study enrollment/index hospitalization and continued until the occurrence of the first endpoint event, death, or last available clinical contact. Follow-up was censored on the date of the last documented follow-up for patients without an event. The final follow-up assessment was completed in January 2026. Overall follow-up time was estimated using time-to-event methodology and is reported as median (IQR). Patients were contacted annually by telephone. For reported events (stroke, TIA, myocardial infarction, atrial fibrillation), the date of diagnosis was recorded and verified by medical documentation. For deceased patients, information was obtained from relatives and primary care physicians.

### Statistical analysis

Statistical analyses were performed using SPSS version 26. Continuous variables are presented as mean ± SD. Between-group comparisons were conducted using the unpaired *t*-test for normally distributed variables and the Mann-Whitney *U* test or Kruskal-Wallis test for non-normally distributed variables, as appropriate. Categorical variables were compared using the chi-square test. Time to first event was used for endpoint analyses. First, outcomes were compared between ESUS patients and controls. The cumulative incidence of the primary and secondary endpoints was estimated using the Kaplan-Meier method, and survival curves were compared with the log-rank test. In a second analysis, ESUS patients with and without the primary endpoint were compared. All variables were first examined in univariable analyses. Parameters significantly associated with the primary endpoint were entered into Cox proportional hazards regression models to identify independent predictors. Multivariable Cox regression analyses were performed using a theory-driven approach. Clinically relevant covariates, including age, sex, hypertension, diabetes mellitus, and renal function, were prespecified and included irrespective of univariable significance based on their established association with cardiovascular outcomes and atrial remodeling. In addition, atrial remodeling variables of primary mechanistic interest were incorporated based on biological plausibility and prior evidence. Given the limited number of outcome events, parsimonious multivariable models with a restricted number of covariates were used to minimize the risk of overfitting and model instability, in accordance with established events-per-variable considerations. Therefore, no additional sensitivity analyses with more extensive covariate adjustment were performed. The proportional hazards assumption for Cox regression models was assessed by visual inspection of log-minus-log survival plots and evaluation of time-dependent effects. No major violations of the proportional hazards assumption were observed for the variables included in the final models. Therefore, no additional model modifications were required. Receiver-operating characteristic (ROC) analyses were performed in an exploratory manner to identify clinically meaningful threshold values for atrial remodeling parameters. Because conventional ROC analyses do not intrinsically account for censoring in time-to-event data, the derived thresholds should be interpreted with caution and considered hypothesis-generating. Identified risk factors were subsequently integrated into a composite risk model categorizing patients according to the presence of <3 vs ≥3 markers. Event-free survival across risk strata was assessed using Kaplan-Meier analyses and compared using the log-rank test. Future studies should validate these thresholds and risk stratification approaches in independent cohorts using dedicated time-to-event prediction methods. A 2-sided *P* < 0.05 was considered statistically significant.

## Results

### Study population

Between July 2022 and December 2024, 1,622 patients with ischemic stroke were treated at our hospital. During this period, all consecutive patients fulfilling ESUS criteria were screened for eligibility. A total of 112 patients met the prespecified inclusion and exclusion criteria. Four patients declined participation and 5 were lost to 1-year follow-up. The final ESUS cohort comprised 103 patients (50 women, 53 men; mean age 70.6 ± 13.3 years). The control group included 123 individuals (59 women, 64 men; mean age 70.0 ± 14.6 years). ESUS and control groups did not differ with respect to age, sex, body mass index, or LVEF (Table 1). Median follow-up was 382 days (IQR: 366-697 days).

**Table 1:** Baseline Characteristics of ESUS Patients and Matched Controls

	ESUS (n = 103)	Controls (n = 123)	*P* Value
Age (y)	70.6 ± 13.3	70.1 ± 14.6	0.788
Women (♀), n (%)	50 (49)	59 (48)	0.875
Body mass index (kg/m^2^)	27.6 ± 5.7	26.5 ± 4.5	0.087
Systolic blood pressure (mm Hg)	140 ± 23	133 ± 21	0.013
Diastolic blood pressure (mm Hg)	78 ± 14	76 ± 12	0.113
Cardiovascular risk factors			
Hypertension, n (%)	81 (79)	60 (49)	<0.001
Diabetes mellitus, n (%)	29 (29)	10 (12)	<0.001
Hypercholesterinemia, n (%)	42 (41)	18 (12)	<0.001
Laboratory			
NT-pro-BNP (pg/mL) (quartiles)	232 (103; 622)	118 (44; 293)	<0.001
GFR (mL/min/1.73 m^2^)	78 ± 24	80 ± 22	0.553
ECG			
Heart rate (beats/min)	72 ± 13	70 ± 11	0.167
P-wave duration (ms)	118 ± 16	116 ± 17	0.447
Advanced interatrial block, n (%)	14 (14)	2 (2)	<0.001
PTFV1 (mV×ms)	4.1 ± 2.5	3.6 ± 2.3	0.139
P-wave axis (°)	49 ± 28	49 ± 22	0.989
P-wave dispersion (ms)	26 ± 17	27 ± 14	0.708
PR interval (ms)	177 ± 35	174 ± 31	0.508
QRS duration (ms)	95 ± 20	95 ± 16	0.919
Echocardiography			
Left ventricular mass index (g/m^2^)	105 ± 31	90 ± 23	<0.001
Left ventricular ejection fraction (%)	57 ± 7	57 ± 5	0.589
E (cm)	73 ± 23	70 ± 19	0.190
A (cm)	88 ± 25	87 ± 23	0.844
DT (ms)	238 ± 70	231 ± 63	0.469
E′(cm)	8.5 ± 2.6	9.4 ± 2.8	0.011
A′ (cm)	11 ± 3	13 ± 2.5	<0.001
S′ (cm)	9.7 ± 2	10.9 ± 2.4	<0.001
PA-TDI (ms)	148 ± 25	138 ± 17	<0.001
LAD (mm)	36 ± 5	34 ± 5	0.003
LAVI (mL/m^2^)	27 ± 11	24 ± 8	0.029
LAEF (%)	50 ± 12	55 ± 9	<0.001
LASr (%)	21 ± 7	24 ± 7	0.001
TAPSE (mm)	26 ± 4	28 ± 4	0.007

Values are mean ± SD, median (IQR), or n (%). Comparisons between groups were performed using *t*-test, Mann-Whitney *U* test, or chi-square test as appropriate.

A = late mitral inflow velocity; A′ = late diastolic myocardial velocity; BNP = N-terminal pro–B-type natriuretic peptide; DT = deceleration time; E = early mitral inflow velocity; E′ = early diastolic myocardial velocity; ECG = electrocardiogram; ESUS = embolic stroke of undetermined source; GFR = glomerular filtration rate; LAD = left atrial diameter; LAEF = left atrial ejection fraction; LASr = left atrial reservoir strain; LAVI = left atrial volume index; NT-pro-PTFV1 = P-wave terminal force in lead V1; PA-TDI = total atrial conduction time measured by tissue Doppler imaging; S′ = systolic myocardial velocity; TAPSE = tricuspid annular plane systolic excursion.

Compared with controls, ESUS patients had a higher prevalence of cardiovascular risk factors, higher NT-proBNP levels, a greater frequency of advanced IAB, and significant differences in multiple echocardiographic parameters, particularly those reflecting diastolic function and LA hemodynamics (Table 1).

### Clinical events

During follow-up, patients in the control group experienced few events: death (n = 2), stroke (n = 0), TIA (n = 1), myocardial infarction (n = 0), and newly detected atrial fibrillation (n = 1) (4/123, 3%). In contrast, events occurred significantly more frequently among ESUS patients (29/103, 28%, *P* < 0.001), including death (n = 6), recurrent stroke (n = 11), TIA (n = 8), myocardial infarction (n = 2), and newly detected atrial fibrillation (n = 7). Four patients experienced 2 events (stroke and TIA, n = 2; stroke and atrial fibrillation, n = 1; stroke and death, n = 1); for the primary endpoint, only the first event was considered. Event rates at 1 year and over the entire study period are presented in Table 2.

**Table 2:** Clinical Events During the First Year and Over the Entire Study Follow-Up

	ESUS (n = 103)	Controls (n = 123)
All-cause death	5/6	2/2
Stroke^a^	9/11	0
Transitory ischemic attack	7/8	1/1
Myocardial infarction	2/2	0
Atrial fibrillation	5/7	1/1

Values are the number of events during the first year/total events during follow-up. Four ESUS patients experienced two events (stroke and TIA, n = 2; stroke and atrial fibrillation, n = 1; stroke and death, n = 1); for the primary endpoint, the first event was analyzed.

TIA = transient ischemic attack; other abbreviation as in Table 1.

a *P* < 0.05.

Notably, 11 ESUS patients sustained recurrent ischemic stroke during follow-up (11/103, 11%). In 9 cases, the recurrent event again fulfilled ESUS criteria, whereas 1 was classified as small-vessel disease, 1 as large-artery atherosclerosis, and 1 as stroke of undetermined etiology. In 7 ESUS patients (7/103, 7%), clinical atrial fibrillation was newly diagnosed during follow-up, with a median time to diagnosis of 320 days (IQR: 102-448 days) after study enrollment. Kaplan-Meier curves illustrate the cumulative incidence of the composite primary endpoint (death, stroke, TIA, myocardial infarction, and atrial fibrillation; Figure 2A) and the secondary endpoint (death and stroke; Figure 2B) in both groups.

**Figure 2:**
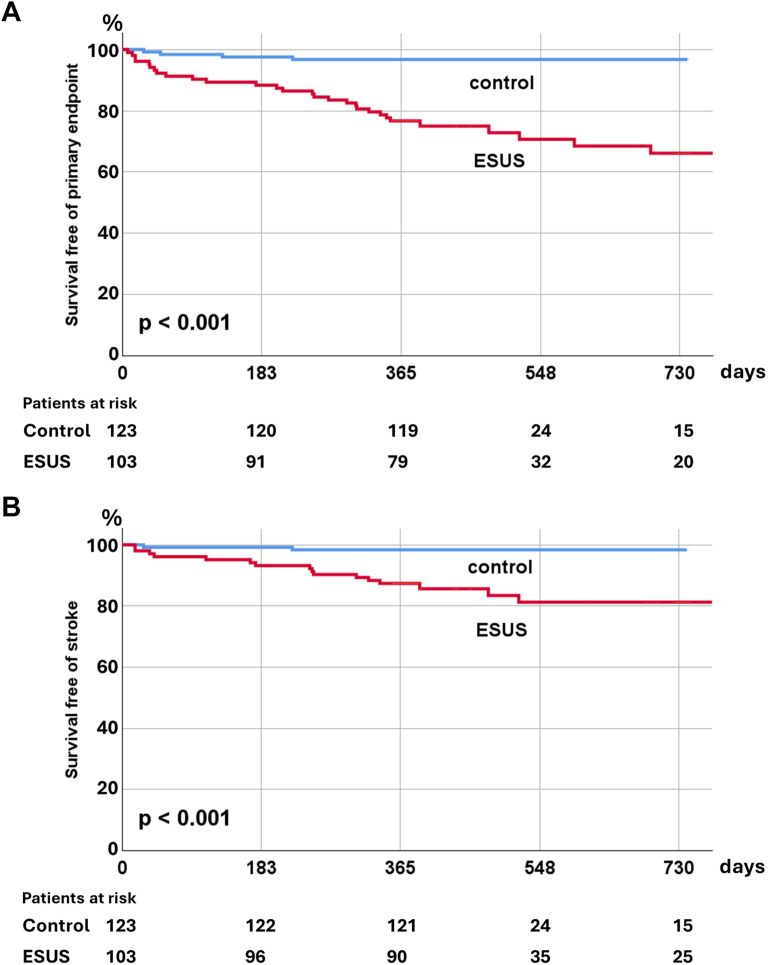
Kaplan-Meier Curves for Embolic Stroke of Undetermined Source Patients and Matched Controls Kaplan-Meier curves illustrating cumulative event-free survival according to embolic stroke of undetermined source status. (A) Cumulative event-free survival for the primary endpoint, defined as the composite of all-cause death, recurrent stroke, transient ischemic attack, myocardial infarction, and newly detected atrial fibrillation. At 1 year, the Kaplan-Meier estimated event rate was 23.3% (95% CI: 15.1%-31.5%) in embolic stroke of undetermined source patients compared with 3.3% (95% CI: 0.2%-6.5%) in controls. Numbers at risk are displayed below the x-axis. (B) Cumulative event-free survival for the secondary endpoint of all-cause death or recurrent stroke. At 1 year, the Kaplan-Meier estimated event rate was 12.6% (95% CI: 6.1%-19.1%) in embolic stroke of undetermined source patients and 1.6% (95% CI: 0%-3.8%) in controls. Numbers at risk are displayed below the x-axis. ESUS = embolic stroke of undetermined source.

### Predictors of the primary endpoint in ESUS patients

Overall, 29 ESUS patients (29/103, 28%) reached the primary endpoint. Compared with those without events, patients with events were older, had a higher burden of cardiovascular risk factors, higher NT-proBNP levels, and significant differences in several hemodynamic and atrial functional parameters (Table 3).

**Table 3:** ESUS Patients With and Without Primary Study Endpoint

	Primary Study Endpoint (n = 29)	No Primary Study Endpoint (n = 74)	*P* Value
Age (y)	76 ± 14	68 ± 12	0.008
Women (♀), n (%)	18 (62)	32 (43)	0.097
Body mass index (kg/m^2^)	27.3 ± 6.4	27.7 ± 5.5	0.751
Systolic blood pressure (mm Hg)	146 ± 23	138 ± 22	0.095
Diastolic blood pressure (mm Hg)	80 ± 15	78 ± 14	0.482
Cardiovascular risk factors			
Hypertension, n (%)	27 (93)	54 (74)	0.031
Diabetes mellitus, n (%)	12 (41)	17 (23)	0.062
Hypercholesterinemia, n (%)	15 (52)	27 (36)	0.172
Laboratory			
NT-pro-BNP (pg/mL) (quartiles)	650 (197; 2,291)	176 (91; 346)	<0.001
GFR (mL/min/1.73 m^2^)	73 ± 28	81 ± 22	0.159
ECG			
Heart rate (beats/min)	72 ± 13	72 ± 13	0.952
P-wave duration (ms)	119 ± 18	117 ± 15	0.533
Advanced interatrial block, n (%)	11 (38)	3 (4)	<0.001
PTFV1 (mV×ms)	3.8 ± 3.2	4.1 ± 2.2	0.509
P-wave axis (°)	51 ± 37	49 ± 25	0.705
P-wave dispersion (ms)	25 ± 17	26 ± 17	0.764
PR interval (ms)	187 ± 46	173 ± 29	0.068
QRS duration (ms)	95 ± 19	95 ± 20	0.939
Echocardiography			
Left ventricular mass index (g/m^2^)	112 ± 35	103 ± 29	0.215
Left ventricular ejection fraction (%)	57 ± 8	57 ± 7	0.902
E (cm)	81 ± 29	70 ± 19	0.029
A (cm)	91 ± 30	86 ± 23	0.411
DT (ms)	245 ± 75	235 ± 68	0.497
E′(cm)	7.6 ± 1.7	8.9 ± 2.8	0.004
A′ (cm)	10 ± 2.8	12 ± 2.9	0.017
S′ (cm)	9.2 ± 2.1	9.9 ± 2	0.099
PA-TDI (ms)	160 ± 29	143 ± 22	0.002
LAD (mm)	37 ± 5	35 ± 5	0.112
LAVI (mL/m^2^)	33 ± 12	24 ± 9	<0.001
LAEF (%)	46 ± 15	52 ± 10	0.017
LASr (%)	18 ± 7	22 ± 7	0.014
TAPSE (mm)	26 ± 4	26 ± 4	0.629

Values are mean ± SD, median (IQR), or n (%). Comparisons between groups were performed using t test, Mann-Whitney *U* test, or chi-square test as appropriate.

Abbreviations as in Table 1.

Variables significantly associated with the primary endpoint in univariable analyses were entered into Cox proportional hazards regression models to identify independent predictors. NT-proBNP, advanced IAB, E′, LA volume index (LAVI), and LA ejection fraction (LAEF) emerged as independent predictors of the primary endpoint.

ROC analyses identified data-derived threshold for NT-proBNP and echocardiographic parameters. For NT-proBNP, the area under the curve (AUC) was 0.746 (95% CI: 0.627-0.866; *P* < 0.001), with a data-derived threshold >420 pg/mL (sensitivity 62%, specificity 78%). For E′, the AUC was 0.649 (95% CI: 0.541-0.757; *P* = 0.007), with a data-derived threshold ≤9 cm/s (sensitivity 76%, specificity 50%). For LAVI, the AUC was 0.735 (95% CI: 0.627-0.843; *P* < 0.001), with a data-derived threshold ≥29 mL/m^2^ (sensitivity 64%, specificity 77%). For LAEF, the AUC was 0.655 (95% CI: 0.518-0.792; *P* = 0.027), with a data-derived threshold <50% (sensitivity 67%, specificity 61%). Corresponding HRs are presented in Table 4.

**Table 4:** Univariate and Multivariate Cox Regression Analyses for Predictors of the Primary Study Endpoint

	HR	CI	*P* Value
Univariate analysis			
Age (y)	1.191	1.026-1.383	0.022
NT-pro-BNP (pg/mL)	1.001	1.000-1.001	0.017
Advanced interatrial block	6.836	3.157-14.804	<0.001
E (cm)	1.018	1.004-1.032	0.010
E′ (cm)	0.827	0.701-0.975	0.024
A′ (cm)	0.848	0.742-0.970	0.016
PA-TDI (ms)	1.022	1.008-1.035	0.002
LAVI (mL/m^2^)	1.053	1.027-1.080	<0.001
LASr (%)	0.937	0.887-0.989	0.018
LAEF (%)	0.963	0.935-0.991	0.010
Multivariate analysis			
NT-pro-BNP ≥420 pg/mL	5.381	2.578-11.233	<0.001
Advanced interatrial block	9.679	4.662-20.096	<0.001
E′ ≤ 9 cm	5.615	1.972-15.987	0.001
LAVI ≥29 mL/m^2^	3.275	1.634-6.656	<0.001
LAEF <50%	3.966	1.863-8.443	<0.001

Values are HRs with 95% CIs and *P* values are shown. The primary endpoint was a composite of all-cause death, stroke, transient ischemic attack, myocardial infarction, or atrial fibrillation.

Abbreviations as in Tables 1 and 2.

### Risk stratification

Based on the 5 independent predictors, a risk score was derived classifying patients into groups with <3 (n = 75), or ≥3 risk factors (n = 28). Kaplan-Meier curves demonstrate increasing event rates across risk strata for the primary endpoint (Figure 3A) and the secondary endpoint (Figure 3B). In patients with ≥3 remodeling markers, Kaplan-Meier estimated event-free survival declined to approximately 50% at 1 year (95% CI: 31.6%-68.4%), indicating a markedly increased cardiovascular risk profile.

**Figure 3:**
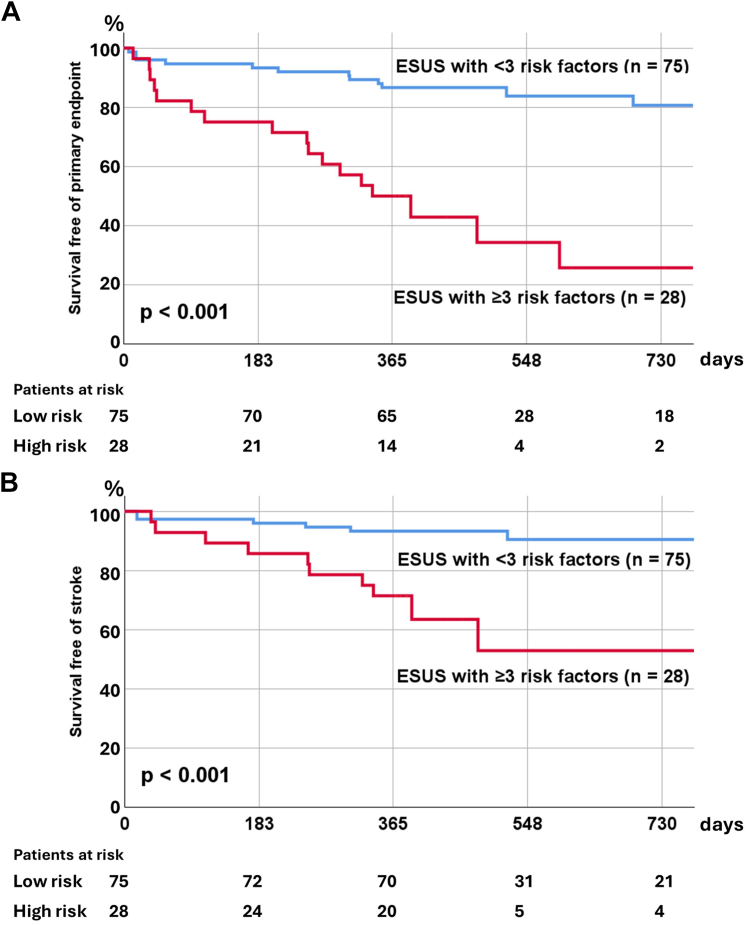
Kaplan-Meier Curves Stratified by Risk Score in Embolic Stroke of Undetermined Source Patients (A) Event-free survival for the primary endpoint (composite of all-cause death, recurrent stroke, transient ischemic attack, myocardial infarction, and newly detected atrial fibrillation) stratified by the number of independent risk factors: <3 vs ≥ 3. Kaplan-Meier estimated 1-year event rates were 50.0% (95% CI: 31.6%-68.4%) in patients with ≥3 remodeling markers and 13.3% (95% CI: 5.7%-20.9%) in patients with <3 markers. (B) Event-free survival for the secondary endpoint (all-cause death and recurrent stroke) stratified by risk score. Patients with ≥3 risk factors (high-risk group) had significant higher event rates compared to patients with <3 risk factors (log-rank test, *P* < 0.001 for both endpoints). Abbreviation as in Figure 2.

## Discussion

### Principal findings

This prospective study provides 3 central insights. First, ESUS patients exhibited a substantial burden of adverse cardiovascular events during follow-up, underscoring persistent residual risk despite contemporary secondary prevention. Second, compared with age- and sex-matched controls without overt cardiac disease, ESUS patients demonstrated consistent evidence of electrical and structural atrial remodeling, supporting the presence of an atrial substrate (AtCM) in a clinically relevant subset. However, the comparison with age- and sex-matched controls should be interpreted as descriptive, as residual differences in cardiovascular risk burden may contribute to the observed differences in atrial parameters. Third, a multimodal combination of biomarker elevation, conduction delay, and impaired atrial mechanics independently predicted adverse outcomes. The presence of ≥3 remodeling markers identified a subgroup of ESUS patients with substantially increased event rates. According to Kaplan-Meier estimates, 14 of 28 patients in this high-risk subgroup reached the primary endpoint within 1 year, corresponding to an estimated event rate of 50% (Central Illustration). These findings extend prior work by integrating multiple domains of atrial remodeling into a unified and exploratory risk stratification approach.

**Central Illustration:**
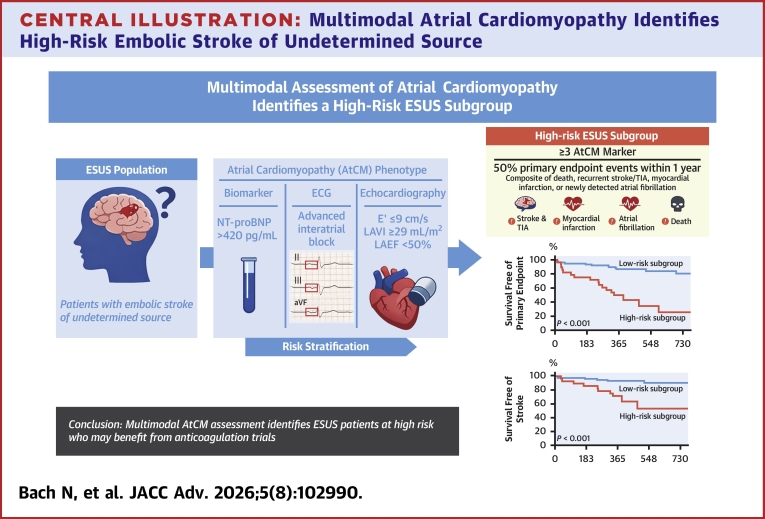
Multimodal Atrial Cardiomyopathy Identifies High-Risk Embolic Stroke of Undetermined Source Patients with embolic stroke of undetermined source exhibited a distinct phenotype of atrial cardiomyopathy characterized by elevated NT-proBNP, advanced interatrial block, and structural left atrial remodeling. Multimodal assessment integrating biomarker, electrocardiographic, and echocardiographic markers enabled risk stratification and identified a high-risk subgroup defined by the presence of ≥3 atrial cardiomyopathy markers. These patients experienced markedly higher rates of adverse cardiovascular events during follow-up, including recurrent stroke/transient ischemic attack, myocardial infarction, newly detected atrial fibrillation, and death. Kaplan-Meier analyses demonstrated significantly reduced event-free survival in patients with ≥3 atrial cardiomyopathy markers compared with those with <3 markers. These findings support a substrate-based approach to risk stratification in embolic stroke of undetermined source and may help identify patients for future rhythm-monitoring or anticoagulation trials. ECG = electrocardiogram; LAEF = left atrial ejection fraction; LAVI = left atrial volume index; TIA = transient ischemic attack; other abbreviation as in Figure 2.

### ESUS as a substrate-driven atrial disorder

ESUS likely represents a heterogeneous entity rather than a uniform pathophysiological condition.^7^^,^^8^ The neutral results of randomized anticoagulation trials in unselected ESUS populations suggest that only a subset of patients harbor a clinically relevant atrial thromboembolic substrate.^9^, ^10^, ^11^, ^12^, ^13^ The present findings indicate that combined electrical and mechanical atrial dysfunction identifies a subgroup of ESUS patients at increased risk of stroke. This supports a substrate-based risk stratification approach and provides a rationale for future randomized clinical trials evaluating anticoagulation strategies in this population.

The concept of AtCM has gained increasing recognition as a mechanistic link between atrial disease and thromboembolism beyond overt atrial fibrillation. Contemporary consensus documents conceptualize AtCM as a progressive continuum of electrical, structural, and functional remodeling capable of promoting thrombogenesis even in sinus rhythm.^7^^,^^8^ However, the identification of clinically meaningful AtCM remains challenging, and validated thresholds for risk-guided therapeutic decision-making are lacking.^6^

Trials such as Apixaban for Treatment of Embolic stroke of Undetermined Source and ARCADIA attempted to enrich ESUS populations using predefined AtCM-related criteria.^11^^,^^12^ Nevertheless, anticoagulation did not reduce recurrent stroke. Importantly, selection strategies relied heavily on isolated markers—particularly NT-proBNP—with limited integration of electrical and mechanical remodeling parameters. Whether these approaches sufficiently captured advanced atrial substrate remains uncertain.

In this context, the present study builds on prior work by combining electrical, structural, and biomarker-based parameters to define a multimodal AtCM phenotype and assess its prognostic relevance. Our findings support and extend the notion that a comprehensive multimodal characterization may better delineate clinically relevant atrial remodeling in ESUS.

### Electrical remodeling as a central determinant

In our study, advanced IAB emerged as one of the strongest independent predictors of adverse events. This ECG marker reflects impaired atrial conduction and electrical dyssynchrony, which are closely linked to mechanical dysfunction and atrial fibrosis.^21^, ^22^, ^23^ Advanced IAB has consistently been associated with atrial fibrillation, thromboembolism, and LA thrombus formation, supporting its role as an electrophysiological expression of atrial disease.^21^, ^22^, ^23^

In the present cohort, advanced IAB was more prevalent in ESUS patients and independently predicted the composite endpoint, highlighting the prognostic relevance of surface ECG markers in substrate-driven risk stratification. These findings reinforce the concept that electrical remodeling may precede or occur independently of documented atrial fibrillation while still conferring thromboembolic risk.

### Structural and functional atrial remodeling

Echocardiographic markers of atrial structure and function further delineated a high-risk phenotype.^24^^,^^25^ ESUS patients exhibited larger LA volumes, reduced atrial ejection fraction, impaired tissue Doppler velocities, and prolonged P-wave to A′ tissue Doppler intervals compared with controls. Among these parameters, LAVI and LAEF independently predicted adverse events, underscoring the importance of mechanical remodeling.

Reduced E′ velocity additionally emerged as an independent predictor, reflecting impaired ventricular relaxation and elevated filling pressures—conditions known to promote atrial remodeling and prothrombotic milieu.^26^ Together, these findings suggest that atrial dysfunction in ESUS is not confined to isolated structural enlargement but represents a broader electromechanical remodeling process.

### Biomarker integration and threshold refinement

NT-proBNP served as a robust and independent predictor of outcome. Although not specific for atrial disease, it reflects myocardial stress and chamber remodeling.^6^ Notably, the optimal cutoff identified in our cohort exceeded previously applied thresholds in enrichment trials, potentially improving specificity for clinically relevant atrial substrate. This observation suggests that refinement of biomarker thresholds may be essential when defining AtCM phenotypes for interventional studies.

### Multimodal phenotyping and risk stratification

A key contribution of this study is the demonstration that integration of electrical, structural, and biomarker-based parameters markedly improves risk discrimination. Approximately one-quarter of ESUS patients (28/103, 27%) exhibited ≥3 remodeling markers, and a half of these patients 14/28 patients (50%) experienced a major cardiovascular event within 1 year.

This multimodal phenotype resembles an “atrial failure–like” state characterized by conduction delay, impaired contractile function, elevated filling pressures, and biomarker activation. Importantly, such substrate characterization may complement existing approaches and provide a more pathophysiologically coherent strategy for future rhythm-guided or anticoagulation trials than reliance on isolated criteria. However, the proposed multimodal score was derived and evaluated within the same data set and was not externally or internally validated. Therefore, the findings may be subject to overfitting and optimism bias and should be interpreted as exploratory.

### Clinical implications

These findings have important implications for arrhythmia-focused risk stratification. First, they support the concept that thromboembolic risk in ESUS may be substrate-driven rather than solely arrhythmia-driven. Second, they highlight the potential value of routine ECG and echocardiographic parameters—widely available and scalable tools—for atrial phenotyping. Third, they suggest that future interventional trials may need to target patients with advanced, multimodal atrial remodeling rather than broad ESUS populations. However, the findings are hypothesis-generating and require validation in independent external cohorts before they can inform clinical decision-making.

### Study Limitations

This was a single-center study with a moderate sample size, which may limit generalizability. Although the cohort was prospectively assembled with strict criteria to minimize competing stroke mechanisms, residual confounding cannot be excluded. Measurements of atrial structure and conduction were performed at a single time point; longitudinal remodeling dynamics were not assessed. The primary endpoint was defined as a composite of death, ischemic stroke, TIA, myocardial infarction, and atrial fibrillation. While this approach increases statistical efficiency in a cohort with limited event numbers, it introduces clinical heterogeneity, as the individual components differ in pathophysiology and relevance to AtCM.

In particular, ischemic cerebrovascular events and atrial fibrillation may be more directly related to the underlying atrial substrate than other components of the composite. Therefore, interpretation of mechanistic associations should be made with caution. Kaplan-Meier analyses and Cox proportional hazards models were used to estimate cumulative event rates and associations with outcomes. Although competing risk methods may provide complementary information for component-specific endpoints, particularly in the presence of death as a competing event, formal competing risk analyses were not performed because of the limited sample size and low number of individual endpoint events. Therefore, component-specific event estimates should be interpreted with caution.

## Conclusions

In ESUS patients, electrical and structural atrial remodeling is frequently observed and is associated with adverse cardiovascular outcomes. A multimodal phenotype incorporating NT-proBNP elevation, advanced IAB, impaired diastolic relaxation, increased LA volume, and reduced atrial ejection fraction identifies a subgroup of patients at particularly high risk, with markedly increased 1-year event rates. These findings extend prior studies evaluating substrate-based risk stratification in ESUS, including approaches used in trials such as ARCADIA and Apixaban for Treatment of Embolic stroke of Undetermined Source.^11^^,^^12^ Collectively, they support a multimodal characterization of AtCM and may help refine risk stratification strategies in ESUS. This approach may support the design of future studies evaluating rhythm-guided or anticoagulation strategies in selected high-risk ESUS populations.

Perspectives **COMPETENCY IN MEDICAL KNOWLEDGE:** Patients with ESUS demonstrate distinct electrical, structural, and functional features of AtCM compared with matched controls. Multimodal assessment integrating biomarkers, electrocardiographic markers, and echocardiographic parameters may improve risk stratification in ESUS beyond clinical characteristics alone. Advanced IAB and elevated NT-proBNP were independently associated with adverse cardiovascular outcomes. A simple multimodal risk score identified an ESUS subgroup with substantially increased 1-year cardiovascular event rates.

**TRANSLATIONAL OUTLOOK:** These findings support a substrate-based approach to risk stratification in ESUS focused on atrial remodeling rather than stroke classification alone. Future prospective studies should evaluate whether multimodal AtCM phenotyping can guide prolonged rhythm monitoring, individualized secondary prevention, or anticoagulation strategies in selected ESUS patients. External validation in larger multicenter cohorts is needed before clinical implementation of the proposed risk model.

## Funding support and author disclosures

Dr Gkizas reports consulting fees, honoraria, and/or speaker fees from 10.13039/100001003Boehringer Ingelheim, 10.13039/100004319Pfizer, and 10.13039/501100005035Biotronik. Dr Haghikia reports consulting fees and/or speaker fees from 10.13039/501100004191Novo Nordisk, 10.13039/100001003Boehringer Ingelheim, 10.13039/100002491Bristol Myers Squibb, 10.13039/100004326Bayer, 10.13039/100000042Amgen, 10.13039/100004336Novartis, and 10.13039/100004319Pfizer. Dr Gotzmann reports consulting fees, honoraria, and/or speaker fees from 10.13039/100006400Alnylam, 10.13039/100001003Boehringer Ingelheim, 10.13039/100002491Bristol Myers Squibb, Eli Lilly, 10.13039/100004336Novartis, and 10.13039/100004319Pfizer. All other authors have reported that they have no relationships relevant to the contents of this paper to disclose.
